# Maternity continuum of care and associated factors among mothers in south Ethiopia: a community-based cross-sectional study

**DOI:** 10.3389/fgwh.2025.1469311

**Published:** 2026-01-30

**Authors:** Leweyehu Alemaw Mengstie, Bethelehem Taye Mengistu, Fekadu Bekele, Wondimagegn Paulos Kumma, Biruk Tesfahun, Yihenew Ayehu Dessie, Wegayehu Zeneb Teklehaimanot, Amanuel Eshetu, Bekahegn Girma, Solomon Abreha, Mohammed Tesema Gebeyehu, Worku Abemie

**Affiliations:** 1 Department of Nursing, School of Nursing and Midwifery, Debre Berhan University, Debre Berhan, Ethiopia; 2 School of Public Health, College of Health Sciences and Medicine School, Welaita Sodo University, Wolaita Sodo, Ethiopia; 3 Department of Nursing, College of Health Sciences, Bulehora University, Bulehora, Ethiopia; 4 Department of Nursing, Hosanna Health Science College, Hosanna, Ethiopia

**Keywords:** associated factors, continuum of care, Ethiopia, maternal health, maternity

## Abstract

**Background:**

The maternity continuum of care (CoC) is crucial for improving maternal and neonatal health outcomes. Despite global initiatives like the SDGs and Every Woman Every Child, maternal health gaps remain in LMICs, especially sub-Saharan Africa, including Ethiopia. This study aims to assess the level of maternity CoC and identify factors associated with its utilization among mothers in Ethiopia.

**Methods:**

A community-based cross-sectional study was conducted between January 15 and February 15, 2024, using a random sample of 564 mothers. Data were collected using a pretested structured questionnaire. Data was entered by EpiData 4.6 and analyzed with SPSS 25. Bi-variable and multivariable logistic regression identified associations, reported with adjusted odds ratios and 95% confidence intervals, with *p*-values <0.05 indicating significance.

**Results:**

Only 23.7% (95% CI: 19.6–27.6) of women completed the maternity continuum of care, highlighting significant gaps in maternal health service utilization. Factors significantly associated with this completion included Secondary and above education (AOR: 5.78, 95% CI: 2.63–12.76), reaching a health facility within 30 min (AOR: 3.71, 95% CI: 1.82–7.57), using family planning services (AOR: 5.13, 95% CI: 2.80–9.39), giving birth at a health facility (AOR: 3.37, 95% CI: 1.97–5.76), and awareness of postpartum complications (AOR: 2.49, 95% CI: 1.36–4.56).

**Conclusion:**

Most mothers did not complete the continuum of maternal care. Key factors for completion of maternal care included secondary and above education, shorter travel times to health facilities, using family planning services, giving birth at health facilities, and awareness of postpartum complications. Enhancing these factors could improve maternal care continuity.

## Background

Continuity of maternity care is an essential program strategy that women have received as a continuation of care throughout the life cycle of pregnancy, childbirth, and postpartum periods and that supports the advancement of women's status and the neonatal health of these women in global health (1).

Globally, in 2020, the maternal mortality ratio was 152 deaths per 100,000 live births, slightly higher than 151 deaths in 2019. This trajectory further projects 133 deaths in 2030, nearly double the SDG target of 3.1 (2). In addition, in 2019, 2.4 million children died in their first month of life. Approximately 6,700 neonatal deaths occur every day. A third of deaths occur within the first 24 h of birth, and three-quarters (75%) occur in the first week of life (3).

In Ethiopia, the magnitude of the continuum of maternity care is 9.1%, according to a multi-level study from 2019 (4).

Studies suggest that a variety of factors influence how often women use different types of maternity healthcare services. Unwanted pregnancies, low educational status, being in the bottom quintile of wealth, not being exposed to the media, living in a rural area, traveling a greater distance to a health facility, being a farmer, starting ANC later than recommended, and not communicating with the husband or other family members were a few of these factors (5–7).

Promoting continuity of care throughout the lifecycle, including adolescence, pregnancy, childbirth, the postpartum period, and childhood, is crucial for maternal, infant, and child health. Continuum of care services helps reduce maternal, neonatal, and pediatric morbidity and mortality (4, 8).

There is currently an updated strategy called “cultivating the culture of completion of the maternity continuum of care,” which states that “each pregnant woman has four or more antenatal care (ANC) contacts, birth is attended by skilled health personnel, and receives early routine postnatal care within two days,” as this will determine the future of women's, neonates', and children's health and lives by 2030 (9).

Despite progress in maternal and child health in South Ethiopia, gaps in the continuum of maternity care persist. Most studies focus on antenatal, intrapartum, or postnatal care separately, lacking a comprehensive approach to coordinated, uninterrupted care from pre-pregnancy to the postnatal period. This study addresses this gap by investigating the factors influencing the seamless provision of maternity care, offering insights to improve maternal and neonatal health outcomes in South Ethiopia.

## Methods

### Study setting, design, and period

A community-based cross-sectional study design was conducted from January 15 to February 15, 2024. The study was conducted in the Damot Gale woreda, Wolaita Zone, South Ethiopia. Currently, there are 29 kebeles in Damot Gale Woreda. Based on the data reported from the Woreda health office, the total population was around 143,720 of those 70,423 were male, with 28,836 households. From the report of Damot Gale Woreda health office, there were 4,836 pregnant women with the first visit of antenatal care, 4,786 women delivered at the health facility and 4,131 women had post-partum care (both health facility delivery and home delivery).

### Population

All mothers in the Damot Gale woreda, Wolaita Zone, South Ethiopia who gave birth within 1 year before data collection were considered the source population.

Selected Mothers gave birth in the last year preceding this study in Gale woreda, Wolaita Zone, South Ethiopia were the study populations.

### Inclusion and exclusion criteria

All mothers who gave birth in the past year in Damot Gale Woreda were included. Moreover, all mothers who gave birth in another district and came to the study area and mothers critically ill and unable to respond to the interview were excluded from the study.

### Sample size and sampling techniques

The sample size is calculated by using a single population proportion formula assuming that the prevalence of maternity continuum of care is 37.2% (10), a 5% marginal error with 95% confidence level, then the sample size is 358, by adding 5% of non-response rate and 1.5 design effect, the final sample size was 562.

The study included 8 Kebeles, chosen by a lottery method (simple random sampling) from 29 Kebeles. Study subjects were allotted to each Kebeles and selected using simple random sampling until the required sample size was met.

### Operational definition

Completion of maternity continuum of care: magnitude of maternity continuum of care was defined as whether a post-partum period woman having one or more ANC visits at the health facility during pregnancy, childbirth aided by SBA (doctor, nurse, and midwife, health officer, and health extension worker), and having one or more PNC for the mothers within 6 weeks after viable childbirth based on self-reports (11).

Completion of Continuum of Care (CoC): It is defined by the completion of all recommended ANC visits (at least 4 ANC), institutional delivery (ID), and PNC services. A woman is said to have completed CoC, coded as “1” if she received all mentioned services, and incomplete CoC, coded as “0”, if she missed at least one of the recommended services. This outcome was used to see the effect of the place of the first ANC visit on the continuum of care (12).

### Study variables

#### Dependent variables

Completion of the Maternity Continuum of Care.

#### Independent variables

Socio-demographic characteristics.

Healthcare service-related factors.

Obstetrical related factors.

**Figure 1:**
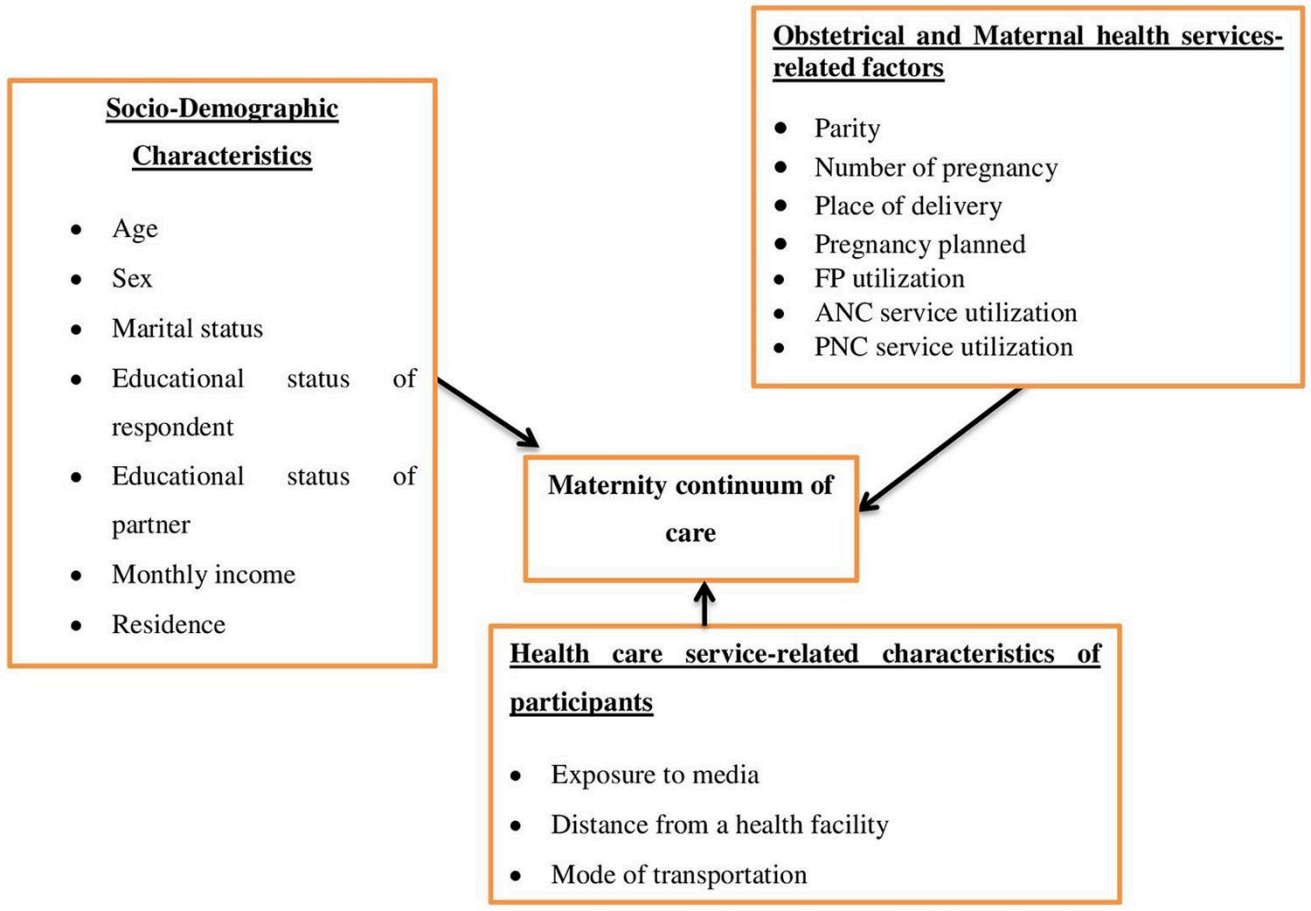
Conceptual frame work for factors associated with continuity of maternal care mothers in Damot Gale Wored Wolaiata Zone south Ethiopia, 2024.

Maternal health care service-related factors (Figure 1).

### Data collection and quality control

Data were collected using a structured interviewer-administered questionnaire developed from the different literature (1–14).

The questionnaire was initially prepared in English, then translated into Wolaytegna (the local language), and back-translated to English to ensure accuracy. The final version was in Wolaytegna for clarity and ease of understanding. Four data collectors (two midwives and two nurses) and two supervisors were recruited for data collection. They received 1 day of training. Principal investigators and supervisors reviewed the collected data daily for completeness and consistency.

### Data processing and analysis

Data was cleaned, coded, and entered into Epidata version 4.6, then analyzed using SPSS version 25. Categorical variables were presented with frequency tables, percentages, pie charts, or bar graphs, while continuous variables were summarized using mean and standard deviation. The hosmer and lemeshow test assessed model fit, and variance inflation factors (VIF) checked for multicollinearity. Bi-variable logistic regression identified variables with *p*-values <0.25 for the final analysis. In the last model, variables with *p*-values <0.05 were deemed significant. Associations were reported using adjusted odds ratios (AORs) with 95% confidence intervals.

## Results

### Socio-demographic characteristics

Out of the 562 samples, 489 mothers participated in the interview, resulting in an 87% response rate. The study found that the average age of respondents was 30.39, with a standard deviation of ±4.07. Out of the respondents, 453 individuals (92.6%) were classified as rural residents. Approximately 148 (30.3%) of mothers and 146 (29.9%) of partners lacked literacy skills. About 196 (40.1%) of mothers were stay-at-home parents and 292 (59.7%) of partners were farmers (Table 1).

**Table 1:** Socio-demographic characteristics of the respondents and their partners in Damot Gale Woreda, Wolaita South, Ethiopia, 2024 (*N* = 489).

Variable	Category	Frequency	Percent
Age of Respondents	15–19	0	0.0
	20–24	29	5.9
	25–29	171	35
	30–34	203	41.5
	≥35	86	17.6
Educational status of a mother	Unable to read and write	148	30.3
	Able to read and write	116	23.7
	Completed primary school	86	17.6
	Completed secondary	72	13.7
	Diploma/above	67	14.7
Educational status of partner	Unable to read and write	146	29.9
	Able to read and write	80	16.4
	Completed primary school	109	22.3
	Completed secondary	104	21.3
	Diploma and above	50	10.0
Marital status of respondents	Married	486	99.4
	Divorced	0	0
	Widowed	3	0.6
Occupation of respondents	Housewife	196	40.1
	Merchant	119	24.3
	Government employee	124	25.4
	Private employee	50	10.2
Occupation of partners	Farmer	292	59.7
	Merchant	165	33.7
	Government employee	18	3.7
	Private employee	14	3.9
Family size	≤5	435	89
	>5	54	11
Distance from health institution	≤30 min	180	36.8
	>30 min but <1 h	190	38.9
	≥1 h	119	24.3

### Maternal healthcare service utilization

**Figure 2:**
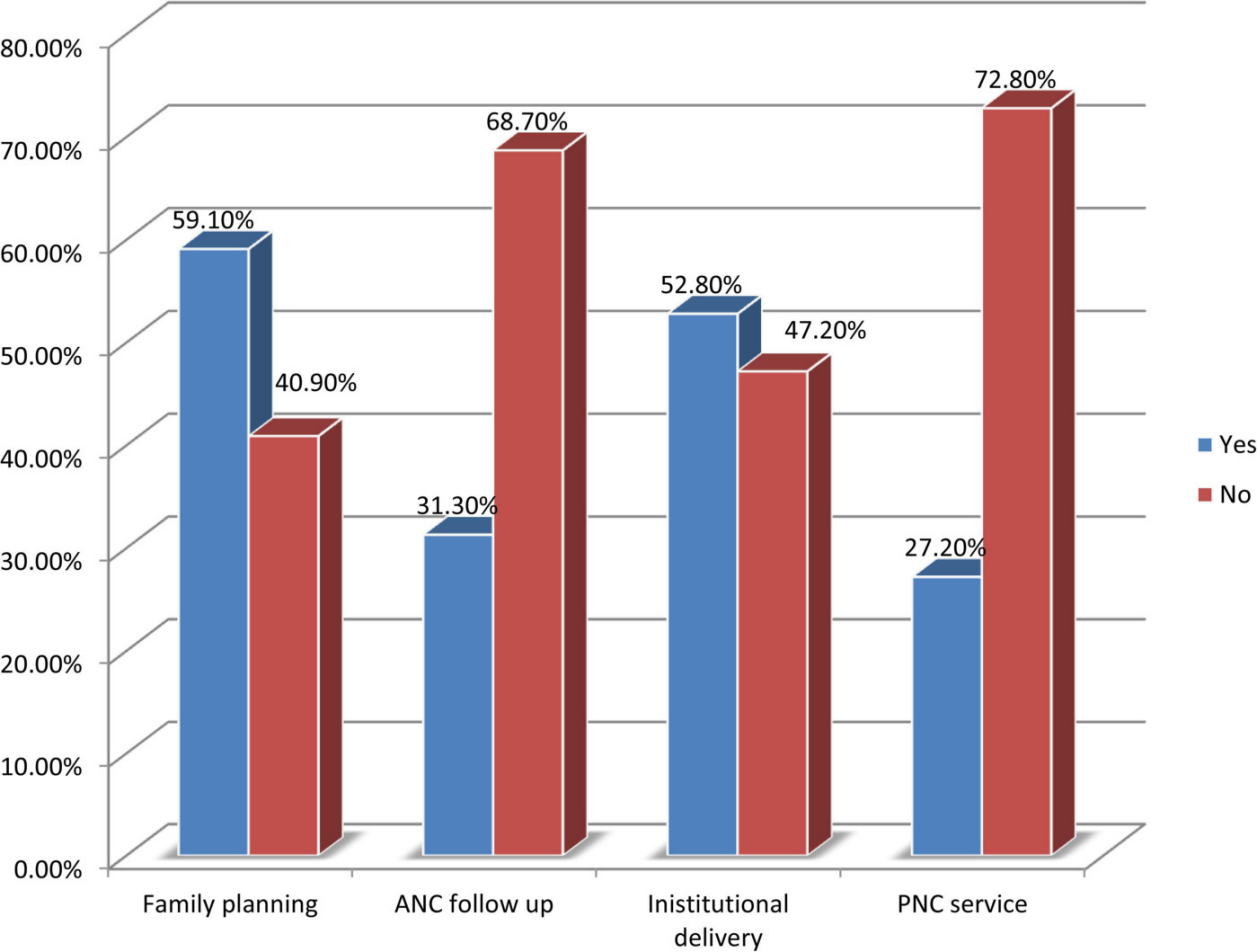
Maternal health care service utilization of the respondents and their partners in Damot Gale Woreda, Wolaita South, Ethiopia, 2024 (*N* = 489).

All respondents have heard about maternal health care (MCH) service and about 289 (59.1%) of respondents use at least one family planning method. About one third 153 (31.3%) of mothers have had attended ANC follow-up at least once for their recent child, almost half 258 (52.8%), and 133 (27.2%) of mothers gave birth at health institutions and have got postnatal care (Figure 2).

**Figure 3:**
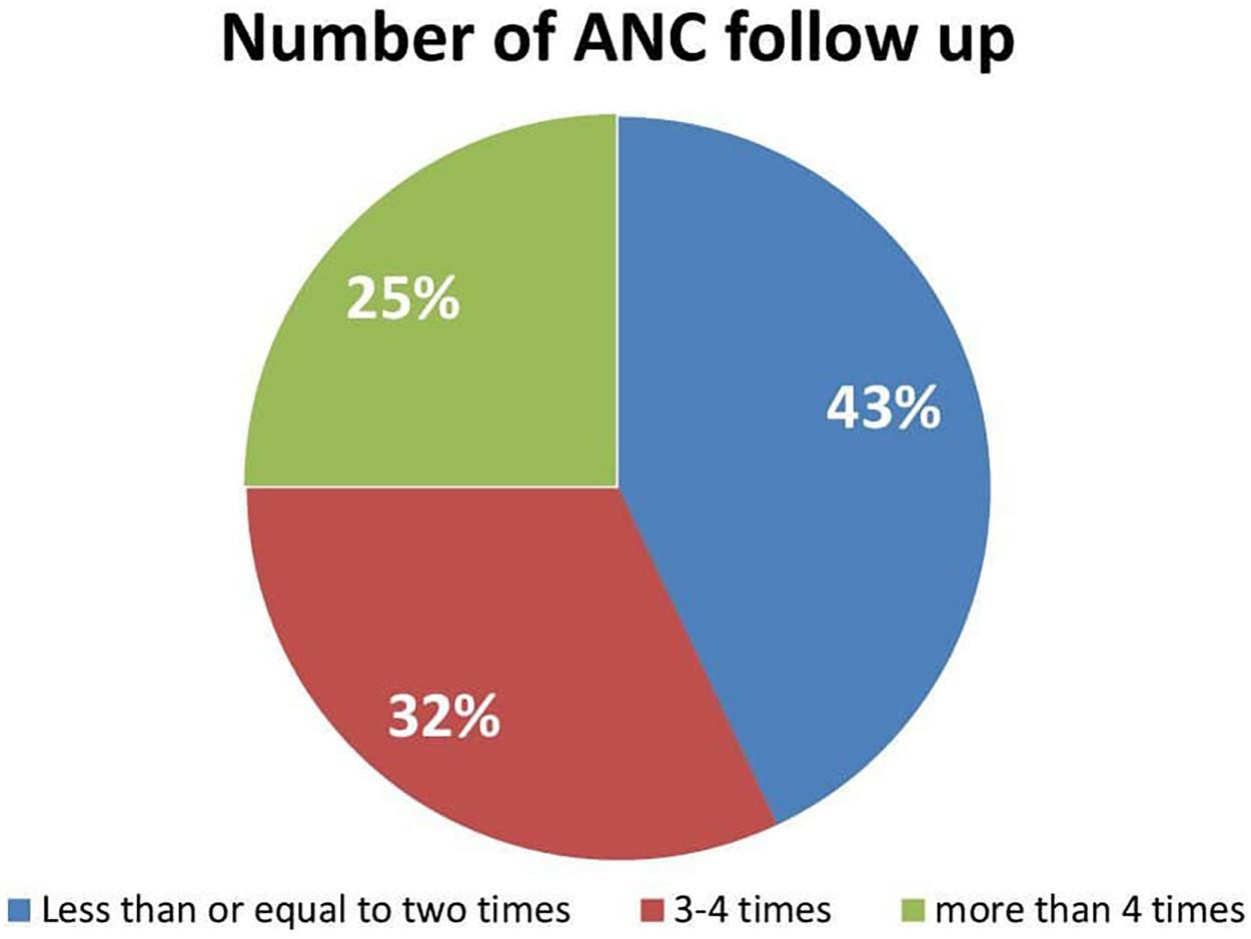
Number of ANC follow up of the respondents in Damot Gale Woreda, Wolaita South, Ethiopia, 2024 (*N* = 489).

Of the total mothers who have had ANC follow up only about one-fourth 38 (25%) followed more than four times (Figure 3).

### Obstetric-related characteristics

Of all the study participants, approximately 58 (11.9%) reported that this was their first pregnancy, while about half, 238 (48.7%), were experiencing their third pregnancy. Nearly all, 483 (98.8%), indicated that the pregnancy was planned, and around 269 (55%) of the mothers had three to four live births (Table 2).

**Table 2:** Obstetric related factors of the respondents in Damot Gale Woreda, Wolaita South, Ethiopia, 2024 (*N* = 489).

Variable	Category	Frequency	Percent
Number of pregnancy	Less than or equal to two	205	41.92
	3–4 pregnancy	238	56.24
	≥4 pregnancy	9	1.86
Number of live birth	≤2 birth	198	40.5
	3–4 birth	269	55.0
	>4 birth	9	1.8
Pregnancy planned	Yes	483	98.8
	No	6	1.2

### Magnitude of continuity of maternity care

**Figure 4:**
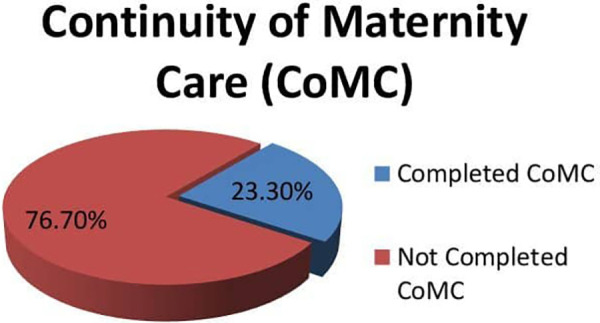
Continuity of maternity care among respondents in Damot Gale Woreda, Wolaita South, Ethiopia, 2024 (*N* = 489).

Of the total respondents who participated in the study, about 116 (23.7%, 95% CI, 19.6%–27.6%) mothers have completed their continuity of care, whereas the remaining 373 (76.3, 95% CI, 72.4%–78.4%) respondents haven't completed their care (Figure 4).

### Factors associated with maternity continuum of care

Variables with a *p*-value of <0.25 in bi-variable logistic regression were candidate variables for the multi-variable logistic regression analysis model. Educational status (completing secondary school and above), time to reach the health facility (less than or equal to 30 min), family planning service utilization, giving birth at a health facility, and counseling about post-partum complications were significantly associated with continuity of maternity care.

Mothers who attended secondary school had 2.46 times higher odds of completing the continuity of maternity care (AOR = 2.46, 95% CI: 1.13–5.37) compared to mothers who could not read or write. Additionally, mothers with above secondary and above education had 3.76 times higher odds of completing the continuity of maternity care (AOR = 5.78, 95% CI: 2.63–12.76) compared to illiterate mothers. Moreover, mothers who took less than 30 min to reach the nearest health institution had 3.71 times higher odds of completing the continuity of maternity care (AOR = 3.71, 95% CI: 1.818–7.57) compared to their illiterate counterparts (Table 3).

**Table 3:** Multivariable logistic regression for factors associated with continuity of maternity care among respondents in Damot Gale Woreda, Wolaita South, Ethiopia, 2024 (*N* = 489).

Variables	Completed CoMC *n* (%)	Not completed CoMC *n* (%)	*p*-value	COR [95% CI]	*p*-value	AOR [95% CI]
Residence						
Urban	13 (36.11%)	23 (33.89%)	0.073	1.92 [0.94–3.93]**		
Rural	103 (22.74%)	350 (77.26%)	1	1		
Maternal educational status						
Can’t read and write	25 (16.89%)	123 (83.11%)	1	1	1	1
Can read and write	22 (18.97%)	94 (87.03%)	0.662	1.15 [0.61–2.17]	0.805	1.10 [0.52–2.32]
Completed primary school	19 (22.09%)	67 (77.91%)	0.327	1.39 [0.72–2.72]	0.320	1.46 [0.69–3.12]
Completed secondary school	21 (29.17%)	51 (70.83%)	0.038	2.03 [1.04–3.94]**	0.023	2.46 [1.13–5.37]***
Diploma and above	29 (43.28%)	38 (56.72%)	<0.000	3.76 [1.97–7.17]**	<0.001	5.78 [2.63–12.76]***
Occupation of respondent						
Housewife	46 (23.47%)	150 (76.53%)	1	1		
Merchant	24 (20.17%)	95 (79.83%)	0.495	0.824 [0.47–1.44]		
Private employee	30 (24.19%)	94 (75.81%)	0.882	1.04 [0.61–1.76]		
Government employee	16 (32%)	34 (68%)	0.217	1.54 [0.78–3.03]**		
Distance from health facility						
≤30 min	68 (37.78%)	112 (62.22%)	1	1	<0.001	3.71 [1.818–7.57]***
>30 min but <1 h	31 (16.32%)	159 (83.68%)	<0.001	3.64 [2.01–6.61]**	0.357	1.42 [0.67–2.99]
≥1 h	17 (14.29%)	102 (85.71%)	0.632	1.170 [0.62–2.22]	1	1
Family planning user						
Yes	97 (33.56%)	192 (64.44%)	<0.001	4.81 [2.83–8.19]**	<0.001	5.13 [2.80–9.39]***
No	19 (9.5%)	181 (90.5%)	1	1	1	1
Place of delivery						
At health facility	171 (66.28%)	87 (33.72%)	<0.001	3.54 [2.22–5.65]**	<0.001	3.37 [1.97–5.76]***
At home	202 (87.45%)	29 (12.55%)	1	1		1
Counseled about post-partum complications						
Yes	95 (27.70%)	248 (72.30%)	0.002	2.28 [1.36–3.83]**	0.003	2.49 [1.36–4.56]***
No	21 (14.38%)	125 (85.62%)	1	1		1

COR, crude odds ratio; AOR, adjusted odds ratio; 1, Reference.

** Significant at *p* < 0.025.

*** Significant at *p* < 0.005.

## Discussion

The study found that the overall completing continuity of maternal care was 23.7% [95% CI, 19.6%–27.6%)], this is comparable with a study conducted in North West Ethiopia at 21.6% (1), a systematic review and meta-analysis conducted in Ethiopia 25.5% (13), Pakistan 27% (15). But, lower than studies reported in some other studies, Debre Berhan 37.2% (10), North West Ethiopia reported completion rates of 37.6% (16, 17), 47% (18), Cambodia (60%) (19) and Egypt (50.4%) (20), and a study in Ghana reported a significantly higher rate of 66% (21).

However, the completion rate observed in this study is higher than those reported in other regions, North East Ethiopia, the completion rate was 11.2% (14), in Arbaminch, it was 9.7% (22), and in Ethiopian Demographic and Health Survey (EDHS) reported a national rate of 6.56% (23), and studies conducted in Ghana found completion rates of 8% a (24).

The disparities in these findings can be attributed to variations in healthcare infrastructure, effective maternal health programs, and higher levels of maternal awareness and education.

According to this study educational status was positively associated with continuity of maternal care which means more educated mothers are more likely to complete continuity of maternal care. Multiple studies conducted worldwide, including our country (2, 25, 26), support this claim. The scientific justification for this is that education enhances health literacy, enabling women to comprehend the benefits of regular healthcare visits and adherence to medical advice (18).

Moreover, mothers who use family planning services were more likely to complete continuity of maternal care compared to their counterparts. This is supported by a study conducted in South Wollo (27), North East Ethiopia (28), and Arbaminch (22). This might be because mothers who use family planning services have a chance to be counseled about ANC, delivery, and PNC services. Due to this may encouraged to complete the continuity of care.

This finding also found that mothers who were near health institutions were more likely to compete for continuity of maternal care compared to mothers who were far away from the health institutions. This finding is consistent with studies conducted in North West Ethiopia (18), North East Ethiopia (27), Gambia (29), and a study conducted in Gahanna (21).

This study also revealed that there is an association between completing continuity of maternal care and giving birth at a health institution as well as being counseled about post-partum complications. This is due to scientifically, counseling increases knowledge and preparedness, reducing the risk of complications (16).

## Strength and limitation

This community-based study, with a large sample size and three home visits, improved response rates. However, recall and social desirability biases may have affected data accuracy.

## Conclusion

The magnitude of completing continuity of maternal care is higher compared to the EDHS report. Significant factors associated with continuity of maternal care include maternal educational status (completing secondary school and obtaining a diploma or higher), using family planning services, giving birth at a health institution, living within 30 min of a health facility, and receiving counseling about post-partum complications.

Therefore, health leaders, policymakers, and all stakeholders should prioritize the continuity of maternal care when providing various services to mothers. Enhancing family planning service utilization, ensuring health facility deliveries, and offering counseling during service provision should be considered to improve the continuity of maternal care.

## Data Availability

The raw data supporting the conclusions of this article will be made available by the authors, without undue reservation.
